# Reducing the Technical Difficulty of Transforaminal Endoscopic Lumbar Discectomy for Upward-Migrated Lumbar Disc Herniation Involving the High Axillar Space: Key Surgical Steps and Proposed Coronal MRI-Based Modified Migration Grading

**DOI:** 10.3390/brainsci16070692

**Published:** 2026-06-30

**Authors:** Dong-Hoon Yang, Mansu Kim, Joong Won Yang, Jae Man Cho, Sang Jin Park, Heum-Dai Kwon

**Affiliations:** Department of Neurosurgery, Pohang Stroke & Spine Hospital, 352 Huimang-daero, Nam-gu, Pohang, Gyeongsangbuk-do 37659, Republic of Korea; pseudo1114@naver.com (M.K.); cgenetor@gmail.com (J.W.Y.); motyuman@naver.com (J.M.C.); chunkook0990@naver.com (S.J.P.); spinekwon@gmail.com (H.-D.K.)

**Keywords:** lumbar disc herniation, upward migration, coronal magnetic resonance imaging, transforaminal endoscopic discectomy, surgical technique

## Abstract

**Highlights:**

**What are the main findings?**

- Standardized key surgical steps, including SAP flattening, detachment of the superior extraforaminal ligament, and cranial rotation with oblique tilting of the endoscope, may facilitate access to upward-migrated LDH involving the high axillar space.
- Lesions involving the high axillar space showed more demanding anatomical characteristics, but favorable early clinical outcomes were maintained.

**What are the implications of the main findings?**

- Standardized key surgical steps may provide a practical framework for accessing upward-migrated LDH involving the high axillar space during the transforaminal endoscopic approach.
- The proposed coronal MRI-based modified migration grading may support preoperative planning by assessing both the vertical height and medial location of upward-migrated LDH and identifying lesions that may require additional surgical maneuvers.

**Abstract:**

Background/Objectives: Upward-migrated lumbar disc herniation (LDH) presents technical challenges during the transforaminal endoscopic approach, particularly when fragments migrate cranially and medially beyond the usual working corridor. This study was based on the hypothesis that surgical difficulty is influenced by both vertical migration height and medial location. We aimed to describe key surgical steps for accessing these difficult lesions and to propose a coronal MRI-based modification of existing migration grading to support preoperative localization and surgical planning. Methods: This retrospective observational cohort study included 66 patients who underwent transforaminal endoscopic lumbar discectomy for upward-migrated LDH. Sagittal MRI-based migration grading was modified on coronal MRI using two reference lines: the inferior pedicle line for vertical grades 1–3 and the inner pedicle line for horizontal grades L and M. Cases with grade 2 or 3 plus grade M were defined as involving the high axillar space (HAS). Patients were divided into non-HAS and HAS groups. Key surgical steps included superior articular process flattening, detachment of the superior extraforaminal ligament, and cranial rotation with oblique tilting of the endoscope. Results: Thirty-five cases (53.0%) involved the HAS. Grade 2 was the most common vertical grade (65.2%), and grade M accounted for 56.1% of cases. Despite more demanding anatomical characteristics, the HAS group achieved early clinical outcomes comparable to those of the non-HAS group, with excellent or good outcomes in 85.7% and 93.6% of patients. Conclusions: Standardized key surgical steps, including SAP flattening, detachment of the superior extraforaminal ligament, and cranial rotation with oblique tilting of the endoscope, may facilitate access to upward-migrated LDH involving the HAS while maintaining favorable early clinical outcomes. The proposed coronal MRI-based modified migration grading may help assess the vertical and medial extent of upward-migrated LDH and support preoperative planning for these technically demanding cases.

## 1. Introduction

Upward-migrated lumbar disc herniation (LDH) is a relatively uncommon form of disc herniation. It typically presents with lateral localization and is frequently associated with foraminal rupture. It is more prevalent in older patients and often occurs in a sequestrated, multi-fragment form, which increases the risk of missed fragments during surgery compared to other types of disc herniation [1,2,3]. Upward migration may extend to the mid-vertebral level [1,4]. Owing to its anatomical location, surgical access is generally more challenging than for centrally located or downward-migrated disc herniation [5,6,7].

Several surgical approaches have been used for upward-migrated LDH, including conventional microscopic posterior approaches, interlaminar or keyhole approaches, translaminar approaches, and transforaminal endoscopic approaches [5,6,7,8,9,10,11]. Posterior or translaminar approaches can provide relatively direct access to highly migrated intracanal fragments, but they may require additional laminar, pars, or facet resection depending on the location of the fragment [5,6]. In contrast, the transforaminal endoscopic approach has the advantage of preserving posterior bony and ligamentous structures and may directly access upward-migrated fragments through the foraminal corridor [8,9,10]. However, its technical difficulty increases when the fragment lies near the pedicle level and beyond the usual transforaminal working corridor. This location can limit visualization and instrument maneuverability, increase the risk of incomplete removal, and often require additional bone work or modified endoscopic trajectories [8,9]. Therefore, a key technical requirement is the creation of a sufficient working corridor that allows safe and reproducible access to the target lesion while the endoscope remains within the epidural space.

Previous grading systems for migrated LDH have primarily classified the craniocaudal extent of fragment migration on sagittal MRI [3,8,12]. These systems are useful for describing migration height and identifying highly migrated fragments; however, they provide limited information regarding the medial location of the ruptured fragment relative to the transforaminal working corridor. During the transforaminal endoscopic approach, surgical accessibility is determined not only by how far the fragment has migrated cranially, but also by whether it extends medially toward the pedicle level and beyond the usual foraminal corridor [8,10,11,12]. Therefore, a grading method based only on sagittal migration height may not fully reflect the technical difficulty of the transforaminal approach. Coronal MRI can assess both the vertical height and medial location of upward-migrated LDH on the same plane.

The present study was based on the hypothesis that surgical difficulty during the transforaminal endoscopic approach for upward-migrated LDH is influenced by both vertical migration height and medial location, and that coronal MRI-based assessment of these two factors may help identify lesions requiring additional surgical maneuvers. Therefore, this study aimed to describe key surgical steps that expand the foraminal working corridor, facilitate safe access, and reduce technical difficulty, and to propose a coronal MRI-based modification of existing sagittal MRI-based migration grading for preoperative localization and surgical planning.

## 2. Methods

This retrospective observational cohort study included patients who underwent transforaminal endoscopic lumbar discectomy for upward-migrated LDH. Surgical accessibility was analyzed using the proposed coronal MRI-based modified migration grading, and the operative procedure was performed according to predefined key surgical steps. The study protocol was approved by the Institutional Review Board (Approval No. PSSH 0475-202312-HR-019-01). The requirement for informed consent was waived by the Institutional Review Board due to the retrospective nature of the study.

### 2.1. Inclusion Criteria

Patients were included based on clinical symptoms and lumbar MRI findings. Inclusion criteria comprised acute or chronic leg pain, sensory disturbance, and motor weakness consistent with radiculopathy and nerve root compression. The diagnosis of upward-migrated LDH was confirmed by lumbar MRI. Surgical levels from L1–2 to L4–5 were eligible.

### 2.2. Exclusion Criteria

Patients were excluded if leg pain was accompanied by symptoms inconsistent with typical radiculopathy, including mismatch with the dermatome level, cutaneous changes (e.g., telangiectasia, reticular veins, or varicose changes), cramping, coldness, morning heaviness, nocturnal symptom aggravation, or chronic leg pain persisting for several years. Cases of upward-migrated LDH in which the ruptured disc was located above the pedicle and the main fragment was situated at the shoulder of the exiting root were treated with an interlaminar approach and excluded from the study. The L5–S1 level was excluded because it has distinct anatomical characteristics and different surgical tactics. Ruptured discs located at the disc space level or showing downward migration were excluded, even if treated with the transforaminal endoscopic approach.

### 2.3. Patients and Clinical Assessment

A total of 66 consecutive patients who underwent transforaminal endoscopic lumbar discectomy for upward-migrated LDH between January 2020 and September 2024 were included. Symptom severity at the initial hospital visit was assessed using the visual analog scale (VAS) [13]. Symptom improvement was evaluated at the first postoperative follow-up visit within one month after surgery and categorized using the modified Macnab criteria [4,14]. Outcomes were defined as follows: excellent, complete or nearly complete resolution of symptoms without activity limitation; good, occasional mild symptoms without significant activity limitation; fair, partial improvement with persistent symptoms requiring medication or additional treatment; and poor, no meaningful improvement, worsening symptoms, recurrence, or need for revision surgery. Postoperative MRI was obtained within one day after surgery to assess residual migrated fragments. Recurrence, persistent symptoms, and subsequent surgery were reviewed retrospectively from the medical records. All procedures were performed by a single experienced spine surgeon using the same operative principles and key surgical steps.

### 2.4. Proposed Coronal MRI-Based Modification of Migration Grading

The gross location of the disc rupture was classified as foraminal, foraminal–paracentral, or paracentral. This referred to the axial location of the main ruptured fragment at the disc level, rather than to the extent of upward migration. The proposed modified grading was intended to provide practical guidance for surgical planning of the transforaminal approach, because ruptured discs located higher and more medially are generally more difficult to access through this route.

**Figure 1:**
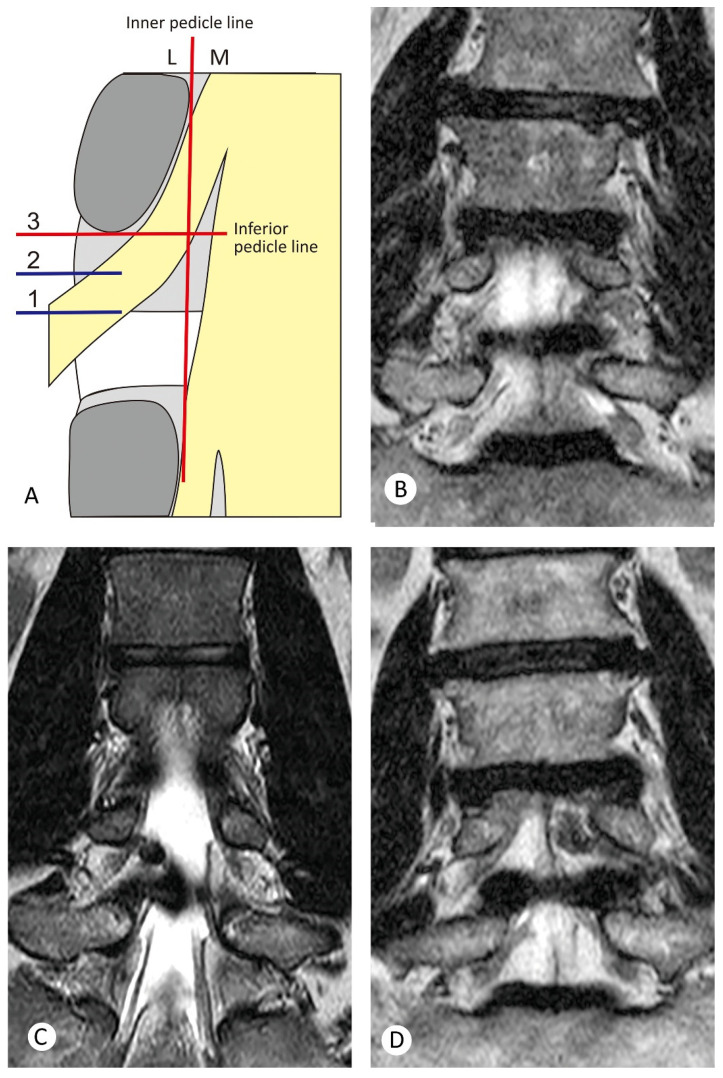
Proposed coronal MRI-based modification of migration grading for upward-migrated LDH. (**A**) Schematic illustration showing grading based on the inferior pedicle line and inner pedicle line. Grade 1: below the midpoint between the upper endplate and inferior pedicle line; Grade 2: above the midpoint but below the inferior pedicle line; Grade 3: above the inferior pedicle line; Grade M: medial to the inner pedicle line; Grade L: lateral to the inner pedicle line. Representative coronal MRI images demonstrate different grades: (**B**) Grade 2L, (**C**) Grade 2M, and (**D**) Grade 3M.

It was defined by two determinants assessed on coronal MRI: the vertical height of the ruptured disc and its medial location. The first determinant, referring to the vertical height of the ruptured disc, was based on previously reported sagittal MRI criteria for the vertical extent of migration [3,8,12]. The original vertical grading concept was maintained, but the terminology was simplified for clarity and ease of interpretation. Grade 1 was defined as below the midpoint between the upper endplate and the inferior pedicle line, grade 2 as above the midpoint but below the inferior pedicle line, and grade 3 as above the inferior pedicle line [8]. The inferior pedicle line served as the reference line (Figure 1).

The second determinant was the medial location of the ruptured disc, assessed using the inner pedicle line connecting the inner borders of the upper and lower pedicles. This line served as the second reference line. Two horizontal grades were introduced accordingly: grade M indicated a ruptured disc located medial to the inner pedicle line, whereas grade L indicated a ruptured disc located lateral to the line (Figure 1).

### 2.5. High Axillar Space

**Figure 2:**
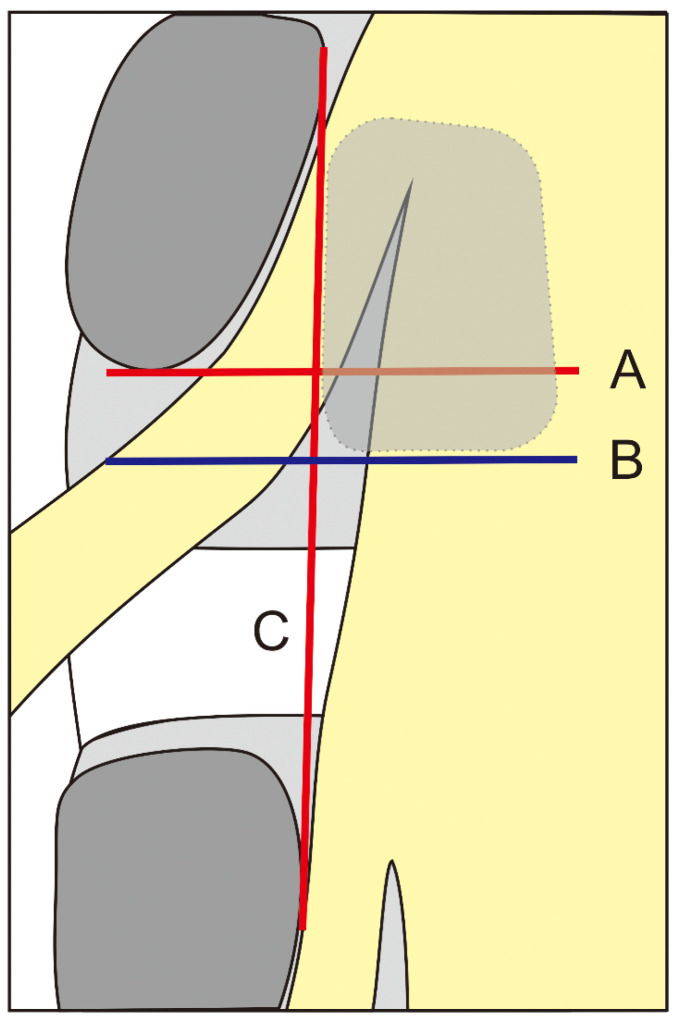
High axillar space (HAS) on coronal MRI. The space is illustrated by the gray dotted area and is bordered medially by the dura mater, laterally by the exiting nerve root, and inferiorly by the midpoint between the upper endplate and the inferior pedicle line. Reference lines are indicated as follows: A, inferior pedicle line; B, midpoint between the upper endplate and the inferior pedicle line; and C, inner pedicle line.

Ruptured discs located at higher positions (grade 2 or 3) and more medially (grade M) were considered more difficult to access through the transforaminal corridor. In this study, this surgically demanding cranial–medial axillar region around the exiting nerve root was termed the high axillar space (HAS). This region is anatomically related to the cranial aspect of the transforaminal working corridor described by Kambin’s triangle, but it is not intended to represent a previously established subdivision of Kambin’s triangle [4]. HAS was defined as the region bordered medially by the dura mater, laterally by the exiting nerve root, and inferiorly by the midpoint between the upper endplate and the inferior pedicle line (Figure 2). Ruptured discs located within this space were assigned to the HAS group, whereas all remaining cases were assigned to the non-HAS group.

### 2.6. Operative Technique

The skin incision and approach angle were determined based on the patient’s back muscle thickness, subcutaneous fat, lumbar level, and, most importantly, the target location identified on axial CT and MRI. Conventional categorizations of transforaminal routes—such as inside-out, outside-in, or extreme lateral—were less relevant in this context.

A trajectory for needle insertion was simulated and marked under C-arm guidance prior to the skin incision. After a small skin incision was made, a needle was advanced toward the lateral bony surface of the superior articular process (SAP) at the mid-disc level. In cases of foraminal narrowing due to foraminal stenosis, the needle tip was directed toward the lower edge of the disc level. The needle was not advanced blindly into the foramen or disc. A guidewire was inserted along the needle pathway, followed by sequential dilators or an obturator over the guidewire. A beveled endoscopic working tube was inserted; a round-end tube was not preferred. The final position of the tube was confirmed at the lateral border of the SAP on the lateral C-arm image. The endoscope was then inserted through the working tube.

**Figure 3:**
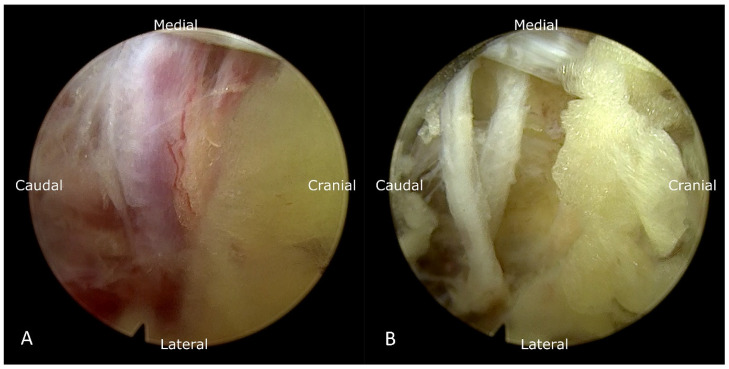
Coagulation of dorsal branches of the segmental vessels during the transforaminal approach. (**A**) Initial endoscopic view showing dorsal branches of segmental vessels at the lateral border of the SAP. (**B**) View after coagulation.

The initial endoscopic view showed muscle fascicles, dorsal branches of the lumbar segmental vessels, and the lateral border of the SAP with the facet capsule. These dorsal branches were a major source of troublesome bleeding during blind insertion of the working tube into the foramen. Under direct endoscopic visualization, they were carefully dissected and coagulated (Figure 3). The dorsal branch of the nerve root and fascicles of the corporotransverse ligament covering the foramen could often be identified.

**Figure 4:**
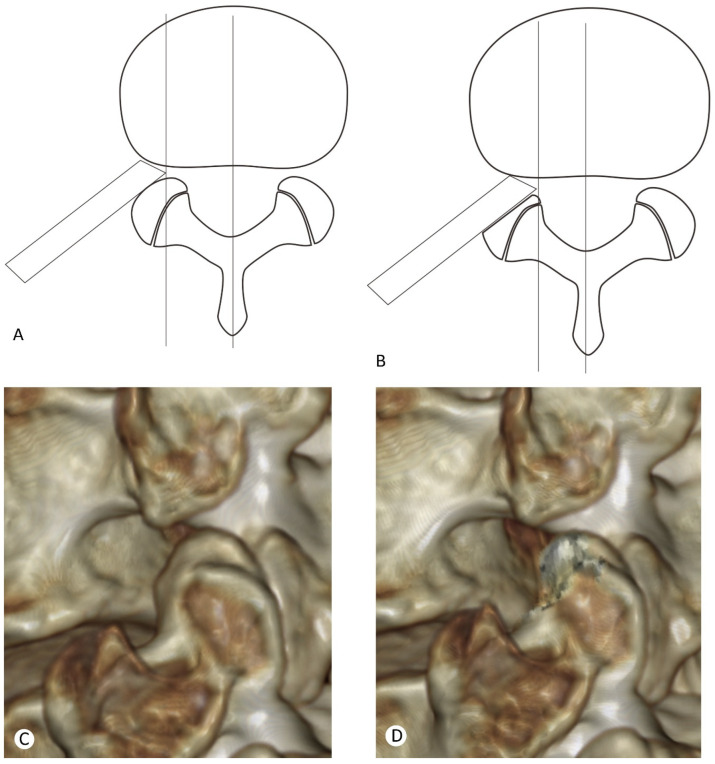
Effect of SAP flattening. (**A**) Convex outer protuberance of the SAP limiting medial advancement of the endoscope before flattening. (**B**) More medial placement after flattening. (**C**) Three-dimensional CT image of the foramen before flattening. (**D**) Increased foraminal size and expanded visualization toward the midline after flattening.

**Figure 5:**
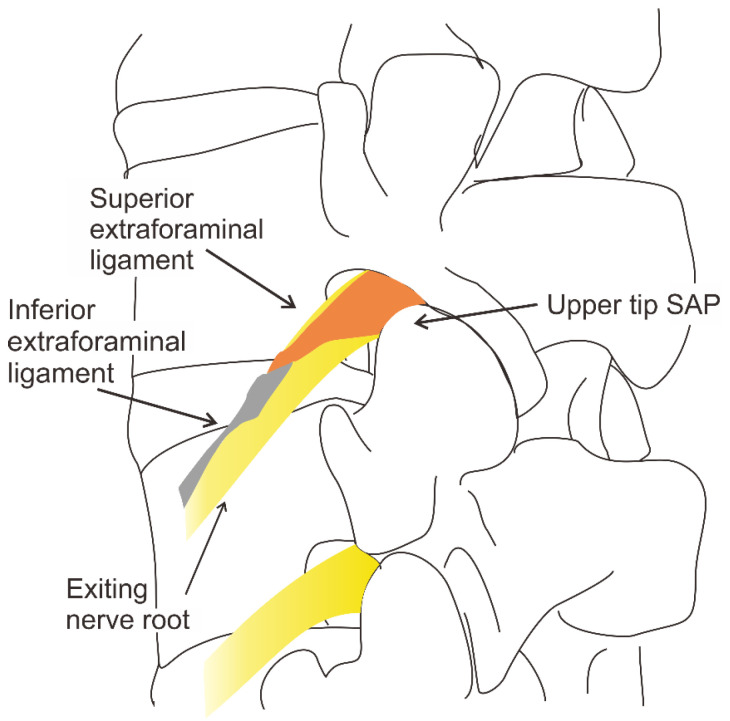
Superior and inferior extraforaminal ligaments viewed from a dorsal oblique angle. The superior extraforaminal ligament forms the roof of the HAS and protects the exiting nerve root outside the foramen.

**Figure 6:**
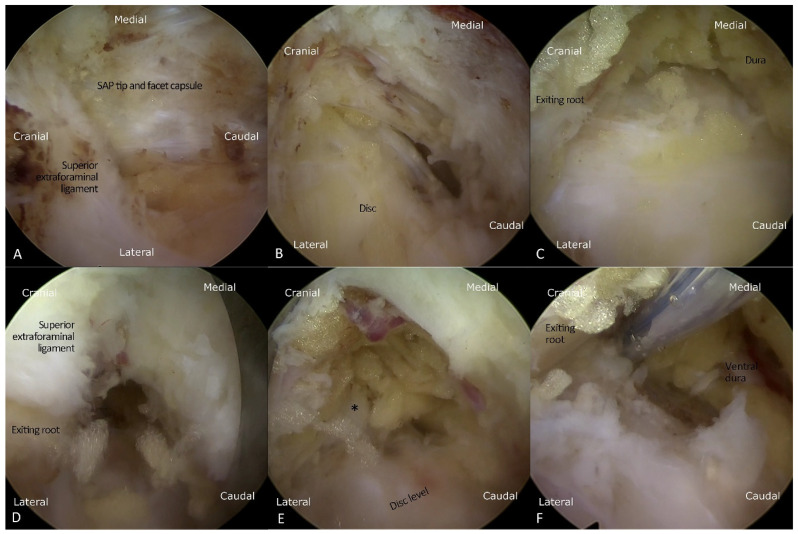
Consecutive endoscopic images of a representative grade 2M case. (**A**) Initial view showing the SAP tip, facet capsule, and superior extraforaminal ligament. (**B**) Opening of the disc space after SAP flattening. (**C**) Exiting nerve root and dura. (**D**) Partial exposure of the HAS after detachment of the superior extraforaminal ligament. (**E**) Ruptured disc fragment (*). (**F**) Decompressed HAS and ventral dura.

**Figure 7:**
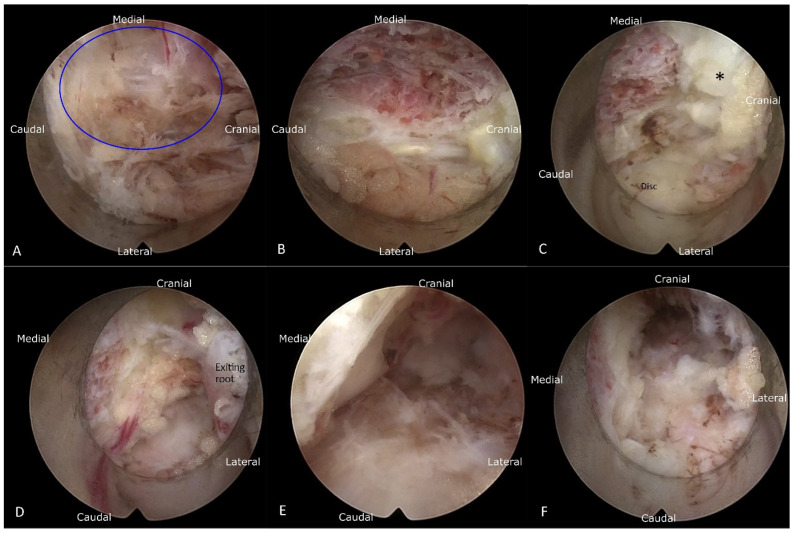
Consecutive endoscopic images of a representative grade 3M case. (**A**) Lateral border of the SAP in the upper half of the endoscopic field (blue circle). (**B**) After SAP flattening. (**C**) Flattened SAP tip and proximal attachment of the superior extraforaminal ligament (*). (**D**) HAS exposed after ligament detachment with further cranial rotation of the endoscope. (**E**) Deeply situated ruptured fragments. (**F**) Decompressed HAS. The cranial direction corresponds to the 3-o’clock position at the beginning of the procedure and is rotated to the 12-o’clock position to access this space.

After clearing the muscles and vessels, the protuberance of the lateral aspect of the SAP became clearly visible. The convex shape of this protuberance may obstruct straight and medial advancement of the working tube, which can increase unnecessary contact with the exiting nerve root. Flattening the SAP protuberance with an endoscopic drill removed this obstacle, allowing a more controlled trajectory to the target. This maneuver represented the creation of a new working space rather than simply exposing an existing space (Figure 4, Figure 5, Figure 6 and Figure 7).

With sufficient flattening of the SAP protuberance, the disc and upper endplate were visualized without retraction of the exiting root. Grade 1 and grade L ruptured discs were removed without further advancement of the instrument.

For access to the HAS, the superior extraforaminal ligament, which functions as the primary protector covering the dorsal roof of the exiting root, was released when necessary. Detaching and cutting its insertion from the facet capsule using cutting forceps or a Kerrison punch expanded the endoscopic view into the HAS. In cases with foraminal stenosis and a thick SAP, resection of the cranial portion of the SAP was also performed when required (Figure 6 and Figure 7).

**Figure 8:**
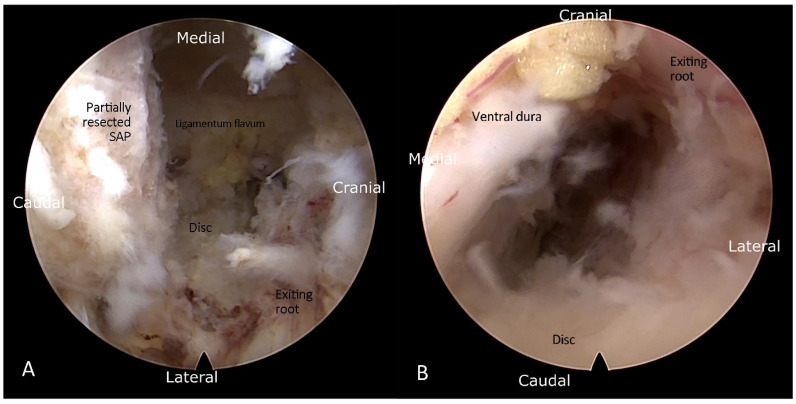
HAS in a case with foraminal stenosis. (**A**) Partial SAP resection up to the disc level. The disc is visible centrally, with the exiting nerve root and ligamentum flavum identified. (**B**) Cranial rotation and tilting of the endoscope reveal the ventral dura and anterior epidural space toward the midline. The exiting nerve root is at the 2-o’clock position and the disc at the 6-o’clock position, while the endoscope remains within the epidural space.

Another key maneuver was cranial rotation and oblique tilting of the endoscope. Because the transforaminal approach uses an oblique trajectory to the target, combining the angled optic of the endoscope with 90-degree cranial rotation and tilting provided substantially expanded visualization of the HAS while the endoscope remained within the epidural space (Figure 6, Figure 7 and Figure 8). During this maneuver, the working cannula was not levered blindly against neural structures. After SAP flattening, the flattened SAP surface and its anterior margin served as a stable bony fulcrum, allowing controlled movement of the working cannula and endoscope toward the HAS under direct endoscopic visualization.

When visualization was insufficient, resection of the superior extraforaminal ligament and the cranial portion of the SAP was extended up to the isthmus. The isthmus served as the final landmark and was preserved, as it functions as a strong structural pillar supporting spinal stability.

As SAP flattening and cutting of the ligament expanded the foraminal working zone, the endoscope maintained adequate mobility within the foramen, enabling more detailed rotation and tilting to maximize target visualization. With the beveled working tube, the exiting root could be gently elevated, allowing visualization of the medio-inferior pedicle margin.

Once the HAS was sufficiently exposed, the ruptured disc was revealed and could be removed without excessive compression of the exiting root. Because upward-migrated ruptured discs are often multi-fragmented, the full extent of fragments observed on coronal MRI was explored and confirmed intraoperatively to avoid leaving residual fragments.

### 2.7. Statistical Analysis

Continuous variables are presented as mean ± standard deviation and were compared using the independent *t*-test. Categorical variables are presented as numbers and percentages and were compared using the Pearson chi-square test or Fisher’s exact test, as appropriate. A *p*-value < 0.05 was considered statistically significant. Statistical analyses were performed using SPSS version 25.0 (IBM Corp., Armonk, NY, USA).

## 3. Results

Among the 66 included cases, 36 patients (54.5%) were men and 30 (45.5%) were women, with ages ranging from 34 to 86 years (mean 63.1 ± 12.3 years). Symptoms affected the right leg in 35 cases (53.0%) and the left leg in 31 cases (47.0%).

Most patients (46 cases, 69.7%) reported symptom duration of less than one month, including 21 cases (31.8%) with symptoms for less than one week. Chronic symptoms lasting more than three months were noted in 7 cases (10.6%). The most affected level was L4–5 (34 cases, 51.5%), followed by L3–4 (25 cases, 37.9%), L2–3 (6 cases, 9.1%), and L1–2 (1 case, 1.5%) (Table 1).

The mean VAS score at the first hospital visit was 8.3 ± 1.0, confirming the severity of symptoms at presentation. This improved to 1.5 ± 0.8 at the first postoperative follow-up visit within one month after surgery. Motor weakness, defined as strength less than grade 4, was observed in 30 cases (45.5%).

Electromyography (EMG) was performed in 61 patients (92.4%), with radiculopathy confirmed in 43 (65.2%). No peripheral neuropathy or other non-radicular findings were detected.

At the first postoperative visit, 53 cases (80.3%) showed excellent symptom improvement, 6 (9.1%) good, 4 (6.1%) fair, and 3 (4.5%) poor outcomes. Among seven fair or poor cases, two underwent fusion surgery within 1–2 years, two experienced recurrent disc herniation within 2–3 months, and three had persistent symptoms requiring long-term medication.

Regarding the gross axial disc rupture location, foraminal rupture was most frequent (44 cases, 66.7%), followed by foraminal–paracentral rupture (21 cases, 31.8%), and paracentral rupture (1 case, 1.5%) (Table 1).

For vertical grading, grade 2 was most frequent (43 cases, 65.2%), followed by grade 3 (12 cases, 18.2%) and grade 1 (11 cases, 16.7%). For horizontal grading, grade M accounted for 37 cases (56.1%) and grade L for 29 cases (43.9%) (Table 2).

Based on whether ruptured discs were located within the HAS, 31 patients (47.0%) were assigned to the non-HAS group and 35 (53.0%) to the HAS group (Table 3).

Age, symptom duration, affected lumbar level, and preoperative VAS score did not differ significantly between the two groups. Motor weakness was more common in the HAS group (19 cases, 54.3%) than in the non-HAS group (11 cases, 35.5%). Foraminal stenosis was also more common in the HAS group (13 cases, 37.1%) than in the non-HAS group (10 cases, 32.3%).

The distribution of vertical migration differed between the two groups. Grade 2 predominated in both groups (non-HAS group: 20 cases, 64.5%; HAS group: 24 cases, 68.6%). Grade 1 was the second most frequent in the non-HAS group (11 cases, 35.5%), whereas grade 3 was second in the HAS group (11 cases, 31.4%). All cases in the HAS group were classified as grade M, whereas only 2 cases (6.5%) in the non-HAS group were grade M (Table 4).

Early clinical outcomes were comparable between the two groups. Excellent or good results were achieved in 29 patients (93.6%) in the non-HAS group and 30 patients (85.7%) in the HAS group (Table 3).

Postoperative MRI obtained within one day after surgery showed no definite residual migrated fragment requiring immediate revision in any patient.

## 4. Discussion

### 4.1. Rationale for Coronal MRI-Based Modification of Migration Grading

Earlier grading approaches for upward-migrated LDH were based mainly on sagittal MRI and focused on the cranio-caudal extent of fragment migration. Although these systems are useful for describing migration height, they provide limited information regarding medial extension and the spatial relationship between the ruptured fragment and the transforaminal working corridor. The proposed coronal MRI-based modification was introduced to help anticipate the technical difficulty of the transforaminal endoscopic approach by integrating two radiologic factors related to accessibility: the vertical height and medial location of the ruptured disc. Coronal MRI was used for this modification, as it can demonstrate the craniocaudal extent and medial spread of the ruptured fragment on the same plane, which may support preoperative localization and surgical planning.

Within-subject comparisons between sagittal and coronal grading were not performed to avoid interpretive coupling. Instead, the distribution of vertical grades in the present study was compared with prior sagittal MRI-based report [8,12]. Grade 2 predominated in the present study (43 cases, 65.2%), whereas Ahn reported a relatively even distribution across grades and Heo found grade 1 to be the most common. These differences suggest that coronal MRI may provide additional information regarding the cranial extent of upward migration, although direct superiority over sagittal MRI cannot be concluded from the present study (Table 2).

Another potential advantage of coronal MRI is its ability to identify the lateral-to-medial spread of the ruptured disc. Using the inner pedicle line as a reference, fragments located medial to this line were positioned farther from the endoscopic entry corridor and were therefore considered more difficult to access than fragments located laterally. By integrating vertical height and medial location, the proposed grading modification may provide a simplified description of the three-dimensional target site. The use of two reference lines, the inferior pedicle line and the inner pedicle line, may help surgeons anticipate surgical difficulty based on proximity to the endoscopic entry corridor (Figure 1).

### 4.2. High Axillar Space

HAS represents a surgically demanding cranial–medial axillar region around the exiting nerve root. In grades 2M and 3M, the ruptured fragment lies farther from the usual transforaminal entry corridor and may be obscured by the SAP, pedicle, and exiting nerve root. Therefore, the difficulty of upward-migrated LDH is not determined only by cranial migration height, but also by whether the fragment extends medially beyond the usual transforaminal working corridor.

The term HAS has not been established in previous publications and is proposed in this study as a surgical-anatomical concept for transforaminal endoscopic approach planning. This concept partially overlaps with Macnab’s hidden zone which broadly describes a surgically difficult subarticular or foraminal region. However, HAS specifically emphasizes the cranial–medial axillar region around the exiting root where an upward-migrated fragment extends toward the pedicle level and medially beyond the usual transforaminal working corridor [4,15].

### 4.3. Extraforaminal Ligament

Anatomical studies of these ligaments are limited, but three layers have been described: the intertransverse ligament, the corporotransverse ligament, and the extraforaminal ligament [16,17,18,19]. Although their morphology varies across reports, the structure most relevant to the transforaminal approach is the extraforaminal ligament. It consists of superior and inferior components that help protect the nerve root from traction-related injury; their proximal attachments maintain the nerve in a central position within the intervertebral foramen and reduce longitudinal tension [16,20,21].

The superior extraforaminal ligament is the larger component and constitutes a major portion of this structure. Its proximal attachment is located at the upper tip of the SAP and the lateral extension of the ligamentum flavum. It forms the roof of the HAS and provides protection for the exiting nerve root outside the foramen (Figure 5).

### 4.4. Key Surgical Steps to Access the High Axillar Space

The rationale of this study was that the addition of standardized key surgical steps may reduce the technical burden of accessing the HAS. Two practical conditions were emphasized: (1) safe and reproducible unroofing of the HAS without reliance on highly individualized techniques, and (2) adequate visualization without excessive retraction of the exiting root. The surgical principles included preservation of key anatomical structures, effective use of the oblique trajectory inherent to the transforaminal approach, and maintenance of endoscopic mobility within the foramen. Particular anatomical attention was directed toward the superior extraforaminal ligament and the configuration of the SAP.

These steps may address different anatomical barriers. SAP flattening improves the medial working trajectory, detachment of the superior extraforaminal ligament unroofs the HAS, and cranial rotation with oblique tilting expands visualization of the craniomedial epidural region while the endoscope remains within the epidural space. Figure 3, Figure 4, Figure 5, Figure 6, Figure 7 and Figure 8 illustrate the key anatomical landmarks and operative maneuvers used to create and maintain access to the HAS.

Although the HAS group exhibited more challenging anatomical features, comparable early clinical outcomes were achieved. This finding suggests that the addition of these surgical steps may help make access to HAS lesions more feasible.

### 4.5. SAP Flattening and Unroofing the High Axillar Space

Foraminoplasty is generally understood as a technique for widening the neural foramen by removing part of the SAP. However, the term has been used broadly in endoscopic spine surgery and may include different targets and extents of SAP resection, such as undercutting of the ventral SAP, resection at the SAP tip, or resection at the SAP base [3,22,23,24]. Therefore, we used the term SAP flattening to emphasize the specific purpose of this maneuver: removing the convex lateral protuberance and anterior tip of the SAP to create a smoother working corridor for medial advancement and cranial rotation of the endoscope toward the HAS.

A subtle but important aspect of foraminal bone anatomy is the convex outer curvature of the SAP, which interferes with medial advancement of the endoscope. Because the exiting nerve root courses obliquely downward through the lateral aspect of the foramen and extraforaminal region, a more lateral endoscopic trajectory may increase the risk of mechanical irritation of the exiting nerve root. In contrast, a more medial endoscopic trajectory toward the center of Kambin’s triangle may help reduce unnecessary contact with the exiting nerve root.

Flattening this convex protuberance with an endoscopic drill allows the endoscope to advance more medially. When flattening is extended to the anterior border and upper tip of the SAP, the hidden foraminal space becomes exposed (Figure 6 and Figure 7). Resection of the entire upper portion of the SAP is unnecessary unless the working corridor is severely compromised by significant foraminal stenosis (Figure 8).

In our technique, drilling is limited to the lateral surface of the SAP, away from the exiting nerve root and dural sac. SAP flattening and foraminal expansion create a sufficient working corridor before deeper advancement, thereby minimizing the need for aggressive levering or excessive exiting nerve root retraction. When removal of the ventral SAP tip near the exiting nerve root is required, it is performed in a controlled manner using an endoscopic Kerrison punch or endoscopic chisel rather than blind drilling. These steps help maintain the working corridor along the bony SAP surface and protect neural structures during access to the HAS.

Detachment and cutting of the proximal attachment of the superior extraforaminal ligament from the SAP is the next key step for expanding the corridor to the HAS. This maneuver allows cranial extension of the unroofing procedure because this ligament lies higher than the tip of the SAP. Together, SAP flattening and ligament detachment create a new working space rather than relying on the narrow natural corridor between the SAP and the exiting root (Figure 6, Figure 7 and Figure 8).

This expanded foraminal working space allows the endoscope to maintain mobility within the foramen and facilitates more precise rotation and tilting of the endoscope to maximize visualization of the HAS (Figure 4). Adequate foraminal widening can also improve visualization of the anterior epidural space and facilitate fragment removal [25,26].

A further advantage of this approach is that the initial landing point of the endoscope is located on the bony surface outside the foramen. This position is safe from unintended nerve root injury and provides a reliable landmark for anatomical orientation. Moreover, the dorsal segmental vessels located on the lateral surface of the facet joint can be dissected and coagulated under direct endoscopic visualization. These vessels are a common source of troublesome bleeding during C-arm-guided blind insertion of the obturator and working tube into the foramen.

For less experienced surgeons, appropriate initial endoscope placement, anatomical orientation, and early bleeding control are major technical barriers. These key surgical steps may help improve procedural safety and operator confidence by providing a more standardized route to the HAS.

### 4.6. Cranial Rotation of Endoscope

**Figure 9:**
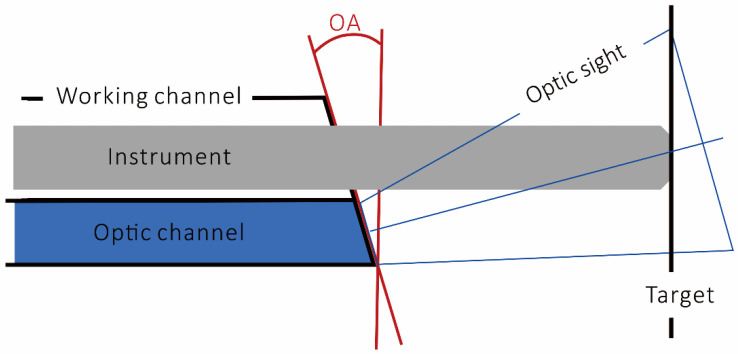
Schematic illustration of the optic angle (OA) and the angled optic field of the endoscope.

A key distinction of the transforaminal endoscopic approach is its oblique angle of access to the target. Whereas most spinal endoscopic approaches follow a vertical trajectory toward a horizontal target, the transforaminal approach takes an oblique path to a horizontal target through the vertical axis of the foramen. When the angled optic of the endoscope is combined with 90-degree cranial rotation and oblique tilting, visualization of the HAS is expanded while the endoscope remains within the epidural space (Figure 8 and Figure 9).

### 4.7. Endoscopic Outcomes in Highly Migrated and Hidden-Zone LDH

Previous endoscopic studies of highly migrated LDH, including lesions located in the hidden zone or similar surgically demanding regions, have reported favorable clinical outcomes but have also emphasized the technical difficulty of standard transforaminal access [8,9,11,15]. These difficulties include limited visualization, restricted instrument maneuverability, anatomical barriers created by the SAP and pedicle, and the risk of incomplete fragment removal. In the present study, excellent or good outcomes were achieved in 85.7% of patients in the HAS group and 93.6% of patients in the non-HAS group, despite the more difficult anatomical characteristics of HAS lesions. These findings suggest that standardized key surgical steps may help facilitate access to HAS lesions, and that the proposed coronal MRI-based modified migration grading may support preoperative identification of cases requiring such maneuvers. The proposed grading modification should currently be regarded as a surgical planning tool rather than an outcome prediction system. Its predictive value and the reproducibility of the operative steps among less experienced surgeons should be evaluated in future prospective studies.

### 4.8. Limitations

This study has several limitations. First, it was a retrospective observational cohort study conducted at a single center, which may limit generalizability. Selection bias could not be excluded because the surgical approach was selected according to clinical and anatomical considerations rather than randomization. Therefore, causal interpretation regarding the effect of the proposed key surgical steps or grading modification on clinical outcomes is limited.

Second, the sample size was relatively small, particularly after division into the HAS and non-HAS groups. No formal sample-size calculation or power analysis was performed because this was a retrospective observational study of consecutive eligible patients. Therefore, non-significant differences between the two groups should not be interpreted as evidence of equivalence, and the findings should be considered exploratory and hypothesis-generating. Multivariate analysis was not performed because of the limited sample size and the small number of unfavorable clinical outcomes after subgrouping, which could result in unstable estimates and overfitting. Therefore, potential confounding factors such as age, affected level, foraminal stenosis, and symptom duration could not be fully controlled. Confidence intervals were also not reported.

Third, the proposed coronal MRI-based modified migration grading was not validated using interobserver or intraobserver reliability analysis and was not formally compared with established migration classifications, including Lee’s classification [11]. Therefore, it should be interpreted as a preliminary surgical planning concept rather than a fully validated classification system. In addition, the value of this grading modification for outcome prediction remains preliminary and requires further validation.

Fourth, no control group treated with conventional microscopic, interlaminar endoscopic, translaminar, or open approaches was included. In addition, a definitive millimeter-based cutoff or coronal zone for selecting an alternative approach instead of the transforaminal endoscopic approach could not be established because this study included only selected candidates for the transforaminal approach and no case required intraoperative conversion due to inaccessible HAS lesions. Therefore, the proposed grading should be interpreted as a surgical planning tool for selected candidates for the transforaminal endoscopic approach rather than as a definitive decision-making algorithm for all upward-migrated LDH cases.

Fifth, although postoperative MRI showed no definite residual migrated fragment requiring immediate revision, radiologic assessment was not performed by independent blinded reviewers; therefore, observer bias cannot be excluded. Quantitative evaluation of foraminal widening or SAP thickness was also not performed.

Sixth, clinical outcome assessment was based mainly on early postoperative VAS and categorical outcome grading at the first postoperative follow-up visit within one month after surgery. Longer-term validated functional outcome measures, such as ODI, were not available for all patients. Therefore, the present findings should be interpreted as early clinical outcomes rather than long-term functional results. In addition, perioperative parameters such as operative time and length of hospital stay were not analyzed because the present study focused primarily on surgical accessibility, technical steps, and early clinical outcomes.

Finally, the results may be influenced by surgeon experience because all procedures were performed by a single experienced spine surgeon. Although this may have improved procedural consistency, it may limit the generalizability of the findings to less experienced surgeons or other institutions. Future prospective multicenter studies are required to validate the proposed grading modification, assess interobserver and intraobserver reliability, compare it with established sagittal MRI-based classifications, evaluate its role in surgical decision-making, compare this approach with alternative surgical techniques, and include longer follow-up with validated functional outcome measures and perioperative variables.

## 5. Conclusions

Upward-migrated LDH involving the HAS is technically demanding during the transforaminal endoscopic approach because the ruptured fragment may extend cranially and medially beyond the usual working corridor. Standardized key surgical steps, including SAP flattening, detachment of the superior extraforaminal ligament, and 90-degree cranial rotation with oblique tilting of the endoscope, may facilitate access to this region while maintaining favorable early clinical outcomes. The proposed coronal MRI-based modified migration grading may help assess the vertical and medial extent of upward-migrated LDH and support preoperative planning for these technically demanding cases.

## Figures and Tables

**Table 1:** Characteristics of symptom manifestation and radiologic findings.

Characteristics	Frequency	Percent (%)
Affected level		
L1–2	1	1.5
L2–3	6	9.1
L3–4	25	37.9
L4–5	34	51.5
Symptom duration		
≤1 week	21	31.8
1–2 weeks	8	12.1
2–4 weeks	17	25.8
1–3 months	13	19.7
3–6 months	2	3.0
>6 months	5	7.6
Type of disc rupture		
Foraminal	44	66.7
Foraminal–paracentral	21	31.8
Paracentral	1	1.5
Total	66	100.0

**Table 2:** Comparison of gradings with previous studies.

Classification	Present Study (%)	Ahn (2022) [12] (%)	Heo (2020) [8] (%)
Vertical grading (height of ruptured disc)			
Grade 1	16.7	32.6	50.0
Grade 2	65.2	32.1	28.6
Grade 3	18.2	35.3	21.4
Horizontal grading (medial location of ruptured disc)			
Grade L	43.9	—	—
Grade M	56.1	—	—
Total	66	47	28

**Table 3:** Comparison of symptoms between the non-HAS and HAS groups.

Variable	Non-HAS (n = 31)	HAS (n = 35)	*p*-Value
Age (years)	62.03 ± 12.54	64.06 ± 12.25	0.510 *
Symptom duration			0.828 ^†^
≤1 week	10 (32.3%)	11 (31.4%)	
1–2 weeks	2 (6.5%)	6 (17.1%)	
2–4 weeks	8 (25.8%)	9 (25.7%)	
1–3 months	7 (22.6%)	6 (17.1%)	
3–6 months	1 (3.2%)	1 (2.9%)	
>6 months	3 (9.7%)	2 (5.7%)	
Affected level			0.216 ^†^
L1–2	0 (0.0%)	1 (2.9%)	
L2–3	2 (6.5%)	4 (11.4%)	
L3–4	9 (29.0%)	16 (45.7%)	
L4–5	20 (64.5%)	14 (50.0%)	
Preoperative VAS	8.29 ± 1.19	8.34 ± 0.87	0.840 *
Postoperative VAS	1.48 ± 0.89	1.57 ± 0.74	0.664 *
Outcome			0.774 ^†^
Excellent	26 (83.9%)	27 (77.1%)	
Good	3 (9.7%)	3 (8.6%)	
Fair ^‡^	1 (3.2%)	3 (8.6%)	
Poor ^§^	1 (3.2%)	2 (5.7%)	

* Independent *t*-test, ^†^ Pearson chi-square test or Fisher’s exact test as appropriate. ^‡^ Fair outcome: In the non-HAS group, one patient underwent fusion surgery after 2 years. In the HAS group, two patients had persistent chronic symptoms, and one patient experienced recurrent LDH within 2 months. ^§^ Poor outcome: In the non-HAS group, one patient experienced recurrent LDH within 3 months with chronic symptoms. In the HAS group, one patient was a revision case with chronic symptoms, and another patient underwent fusion surgery after 1 year.

**Table 4:** Comparison of radiologic findings between the non-HAS and HAS groups.

Variable	Non-HAS (n = 31)	HAS (n = 35)	*p*-Value
Type of disc rupture			<0.001 *
Foraminal	30 (96.8%)	14 (40.0%)	
Foraminal–paracentral	1 (3.2%)	20 (57.1%)	
Paracentral	0 (0.0%)	1 (2.9%)	
Vertical grading			<0.001 *
Grade 1	11 (35.5%)	0 (0.0%)	
Grade 2	20 (64.5%)	24 (68.6%)	
Grade 3	0 (0.0%)	11 (31.4%)	
Horizontal grading			<0.001 *
Grade L	29 (93.5%)	0 (0.0%)	
Grade M	2 (6.5%)	35 (100%)	

* Pearson chi-square test or Fisher’s exact test as appropriate.

## Data Availability

The data presented in this study are available on request from the corresponding author due to privacy and ethical restrictions involving clinical patient data.
